# Supplementary material for: Multimodal factor evaluation system for organismal transparency by hyperspectral imaging
Source: PLoS One. 2023 Oct 11;18(10):e0292524. doi: 10.1371/journal.pone.0292524 (PMC10566722; doi:10.1371/journal.pone.0292524)
Supplement: S1 Table — (PDF) [file pone.0292524.s001.pdf]

S1 Table.

Table S1. The Bio-transparency of the asciidian eggs in various conditions.

environments		Bio-transparency		Relative Attenuation coefficient		fluctuations of bio-transparencies	n (eggs number)	inhibitors		Bio-transparecy		Relative Attenuation coefficient		fluctuations of bio-transparencies	n (eggs number)
Temperature	4°C	89.4% ±	0.9%	0.110 ±	0.01	4.3%	7	Inhibitors	S.W.	88.1% ±	1.0%	0.127 ±	0.01	0.0%	8
	13°C	88.3% ±	1.2%	0.124 ±	0.01	3.2%	7		2DG	89.4% ±	1.9%	0.117 ±	0.02	1.2%	9
	20°C	85.1% ±	1.7%	0.162 ±	0.02	0.0%	10		Mannitol	89.1% ±	2.6%	0.116 ±	0.03	1.0%	9
	27°C	79.5% ±	2.0%	0.236 ±	0.03	−5.5%	10		Actinomycin	89.0% ±	3.8%	0.123 ±	0.04	0.9%	8
	30°C	80.2% ±	1.1%	0.228 ±	0.01	−4.9%	9		Ethanol	87.7% ±	1.9%	0.129 ±	0.02	−0.4%	10
Salinity	0 ppt	65.6% ±	2.1%	0.289 ±	0.02	−18.3%	10		DMSO 0.5%	87.1% ±	4.6%	0.147 ±	0.08	−1.0%	9
	8 ppt	71.7% ±	2.2%	0.247 ±	0.02	−12.2%	7		NaCl	84.1% ±	5.6%	0.175 ±	0.07	−4.0%	6
	20 ppt	78.6% ±	3.0%	0.222 ±	0.04	−5.2%	10		Cycloheximide	83.9% ±	2.2%	0.180 ±	0.03	−4.2%	11
	33 ppt	83.9% ±	1.6%	0.176 ±	0.02	0.0%	8		Oligomycin	82.3% ±	1.8%	0.186 ±	0.02	−5.8%	9
	41 ppt	87.8% ±	1.2%	0.143 ±	0.01	4.0%	8		ML-7	57.3% ±	4.1%	0.496 ±	0.07	−30.8%	14
	55 ppt	34.1% ±	8.1%	1.175 ±	0.26	−49.8%	12		Dead Eggs	36.4% ±	0.6%	0.859 ±	0.01	−51.7%	7
pH	pH1.3	18.2% ±	1.7%	1.811 ±	0.10	−66.4%	8		DMSO 10%	34.7% ±	3.5%	1.136 ±	0.10	−53.4%	9
	pH2.3	20.7% ±	2.6%	1.752 ±	0.12	−63.9%	10								
	pH3.4	87.6% ±	2.4%	0.136 ±	0.03	3.0%	9								
	pH 5.73	88.1% ±	2.6%	0.128 ±	0.03	3.5%	8								
	pH8.06	84.6% ±	3.2%	0.168 ±	0.04	0.0%	6								
	pH10.7	74.8% ±	7.5%	0.297 ±	0.11	−9.8%	8								
