# Supplementary material for: Multimodal factor evaluation system for organismal transparency by hyperspectral imaging
Source: PLoS One. 2023 Oct 11;18(10):e0292524. doi: 10.1371/journal.pone.0292524 (PMC10566722; doi:10.1371/journal.pone.0292524)
Supplement: S2 Table — (PDF) [file pone.0292524.s002.pdf]

# S2 Table.

Table S2. The relative expansion rate of the ascidian eggs in various conditions.

| environments | | relative expansion rate | inhibitors | | relative expansion rate |
|---|---|---|---|---|---|
| Temperature | 4°C | 1.019 | Inhibitors | S.W. | 1.000 |
| | 13°C | 1.001 | | 2DG | 0.965 |
| | 20°C | 1.000 | | Mannitol | 1.000 |
| | 27°C | 0.972 | | Actinomycin | 0.953 |
| | 30°C | 0.966 | | Ethanol | 1.024 |
| Salinity | 0 ppt | 1.465 | | DMSO 0.5% | 0.945 |
| | 8 ppt | 1.349 | | NaCl | 0.996 |
| | 20 ppt | 1.086 | | Cycloheximide | 0.977 |
| | 33 ppt | 1.000 | | Oligomycin | 1.048 |
| | 41 ppt | 0.911 | | ML-7 | 1.127 |
| | 55 ppt | 0.938 | | Dead Eggs | 1.175 |
| pH | pH1.3 | 0.944 | | DMSO 10% | 0.936 |
| | pH2.3 | 0.903 | | | |
| | pH3.4 | 0.983 | | | |
| | pH5.73 | 0.996 | | | |
| | pH8.06 | 1.000 | | | |
| | pH10.7 | 0.992 | | | |
