# Supplementary figures and images for: Multimodal factor evaluation system for organismal transparency by hyperspectral imaging
Source: PLoS One. 2023 Oct 11;18(10):e0292524. doi: 10.1371/journal.pone.0292524 (PMC10566722; doi:10.1371/journal.pone.0292524)

# S1 Fig.

a

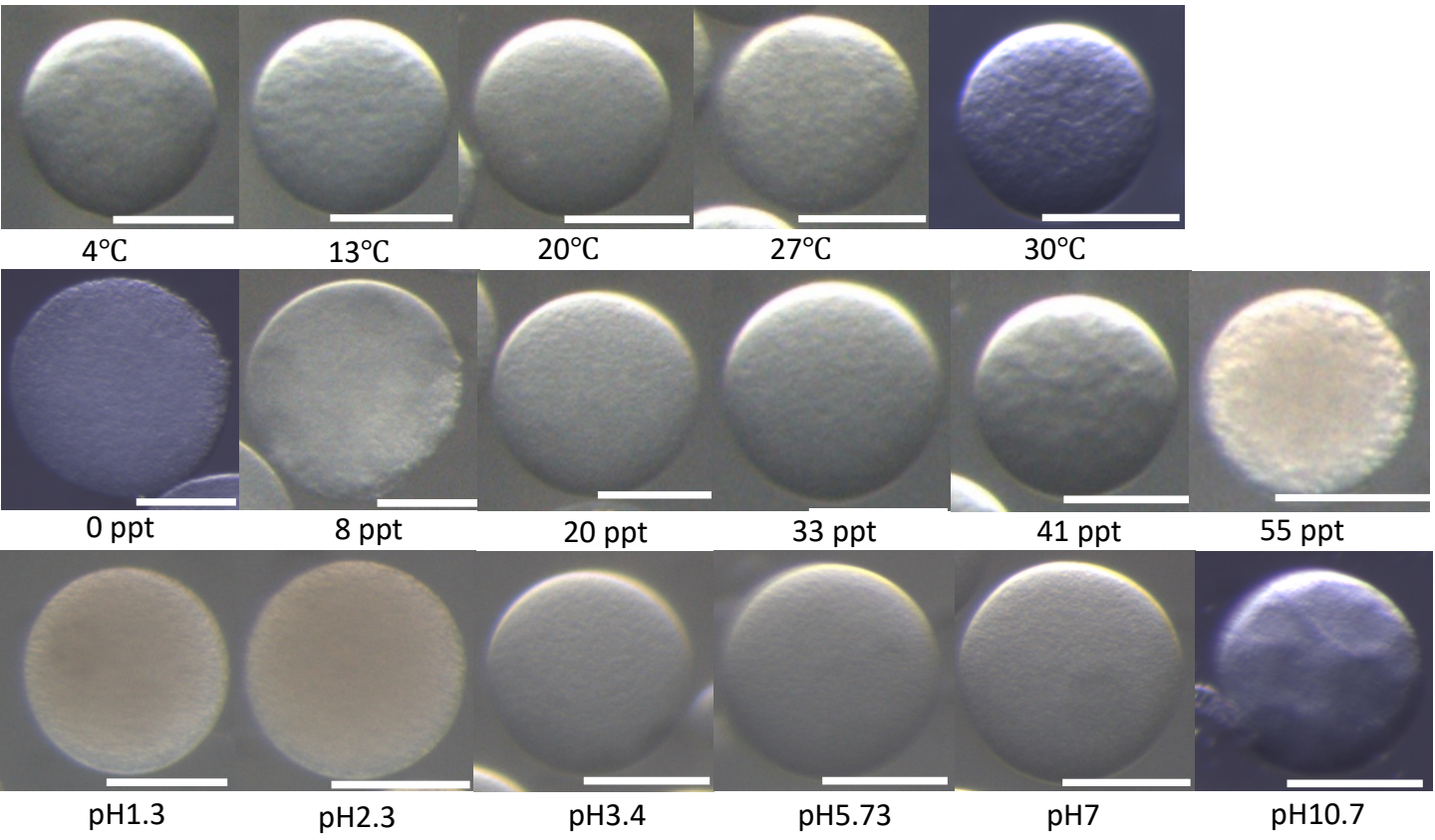

b

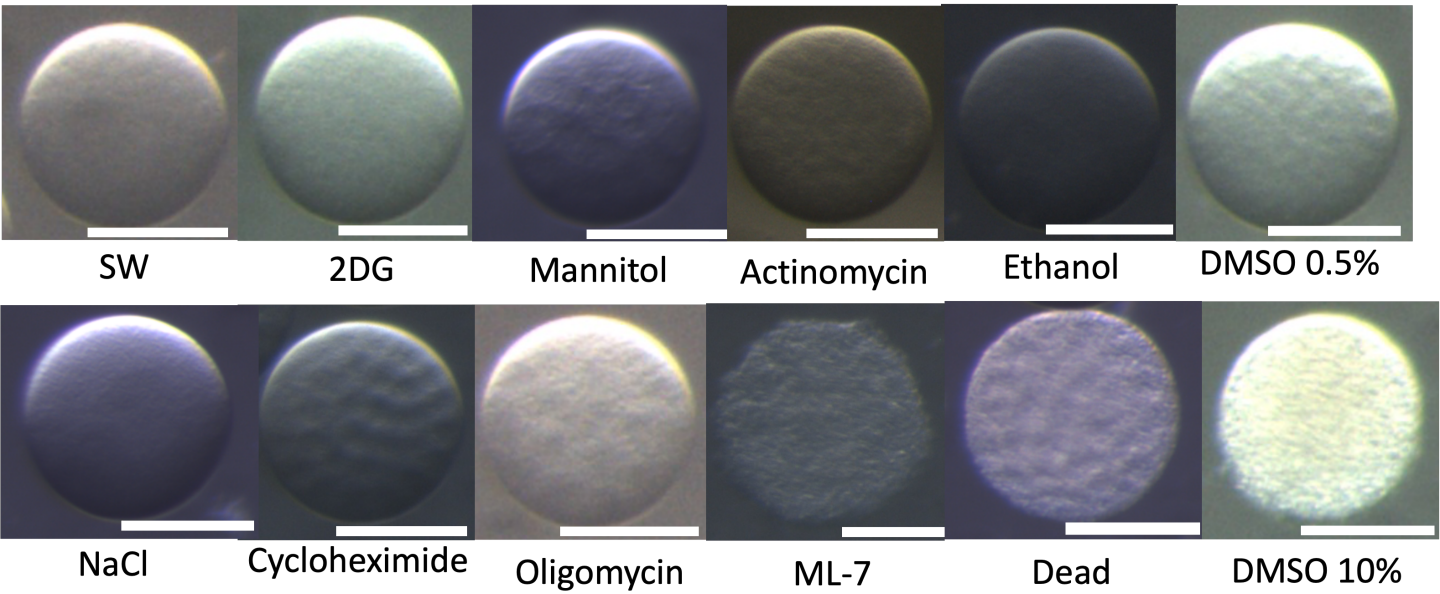

Supplement: S1 Fig — (PDF) [file pone.0292524.s003.pdf]

# S2 Fig.

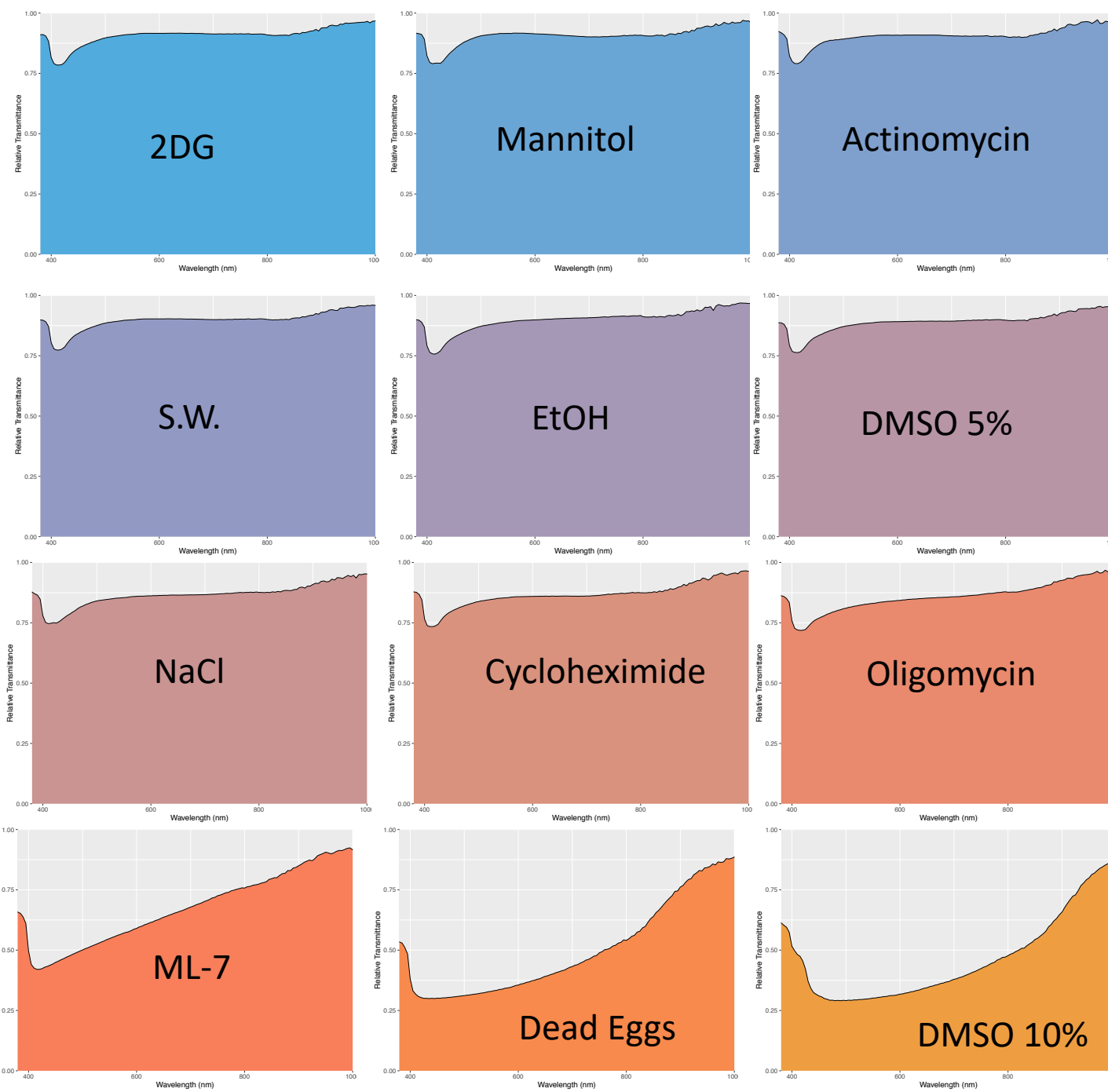

Supplement: S2 Fig — (PDF) [file pone.0292524.s004.pdf]

S3 Fig.

a

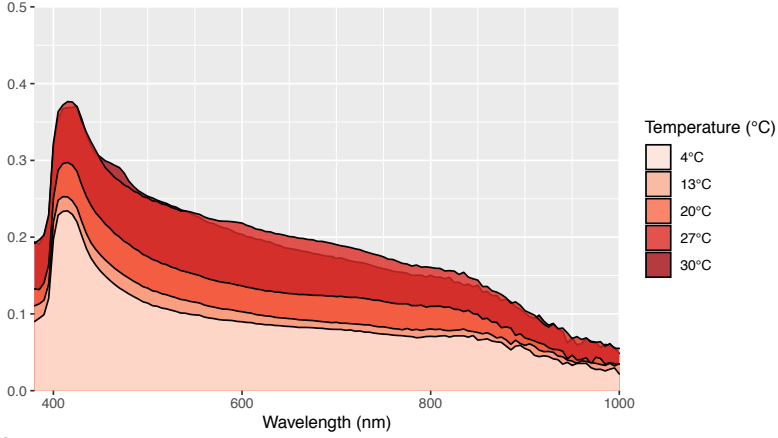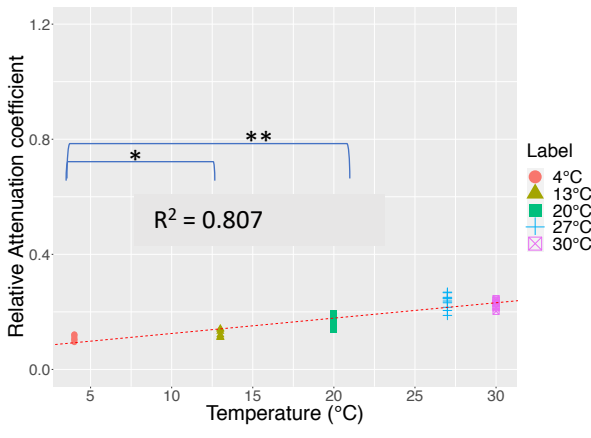

b

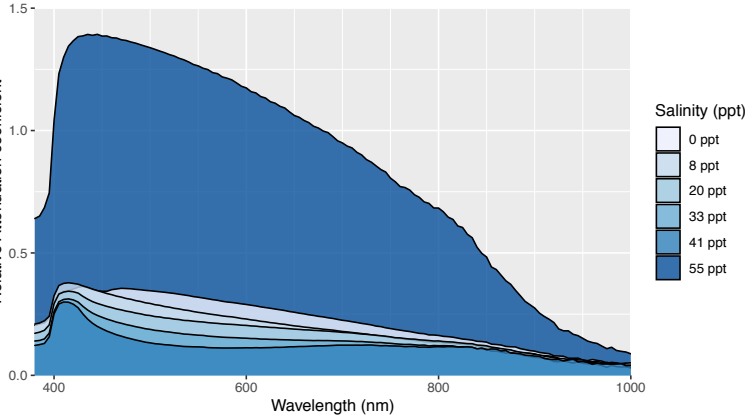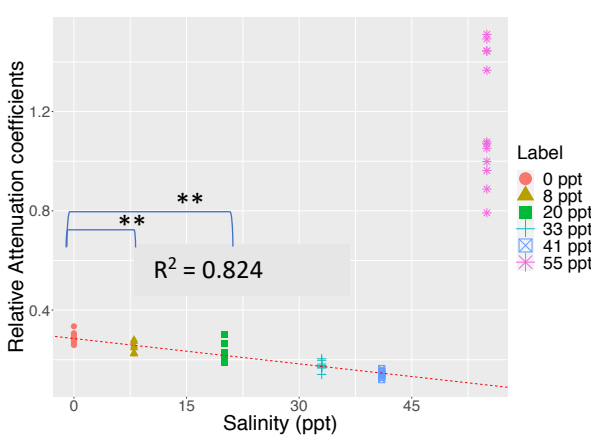

c

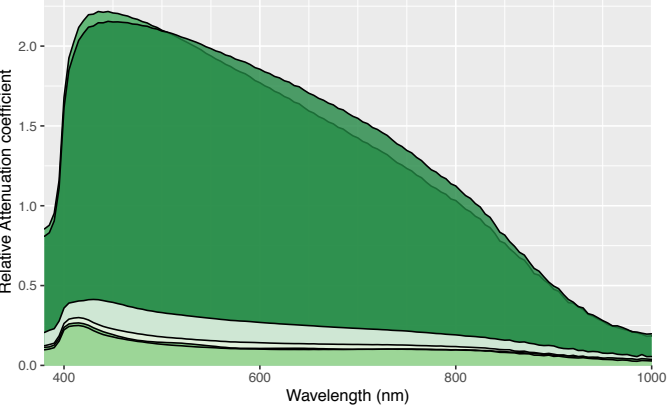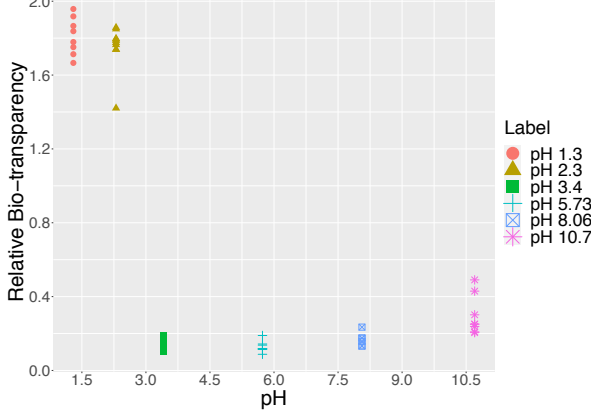

Supplement: S3 Fig — (PDF) [file pone.0292524.s005.pdf]

S4 Fig.

a

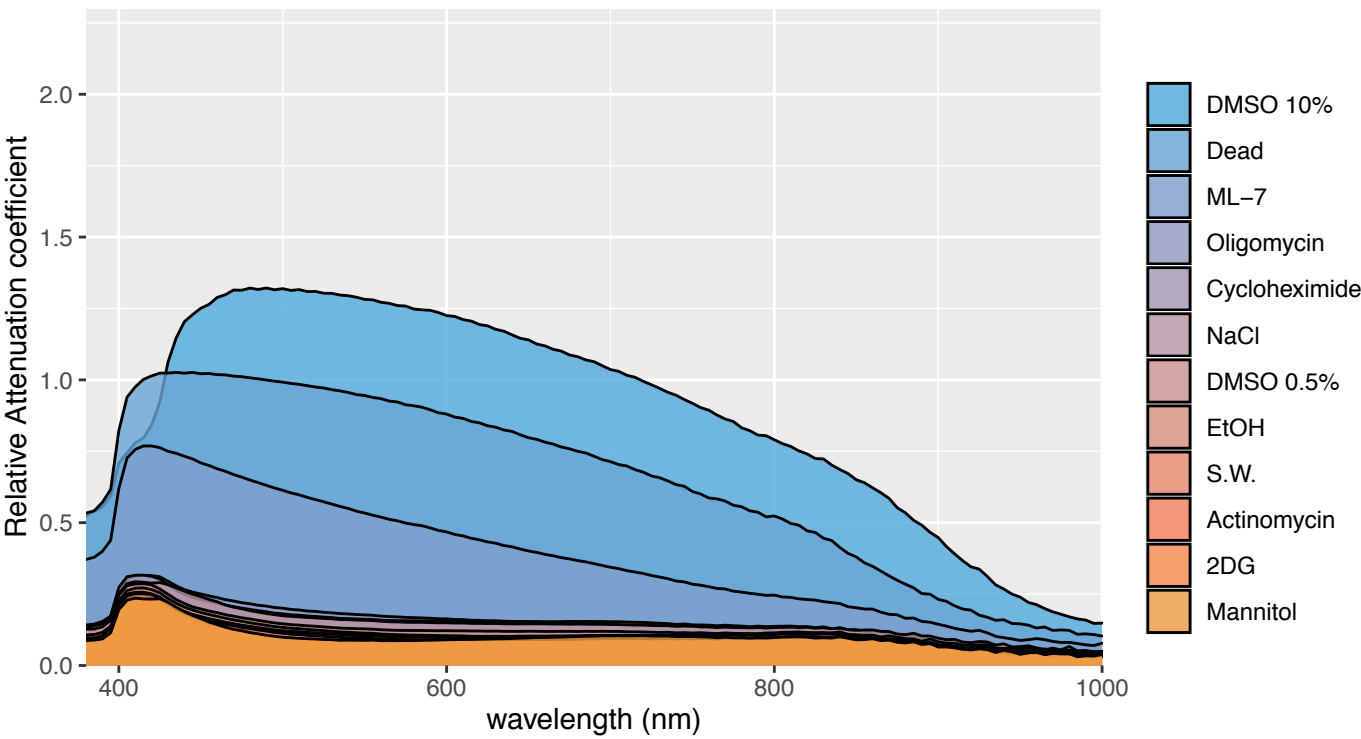

b

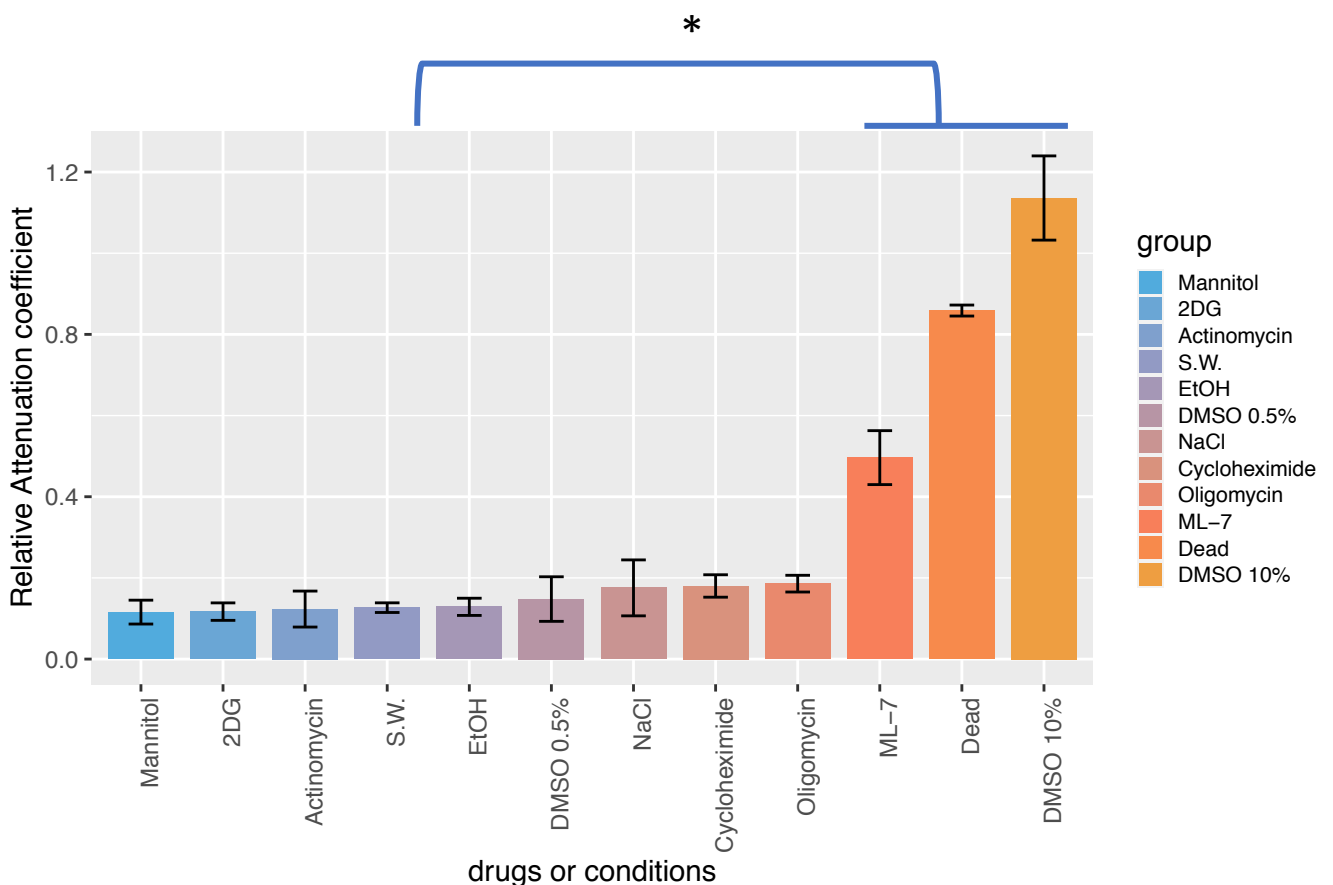

Supplement: S4 Fig — (PDF) [file pone.0292524.s006.pdf]

S5 Fig.

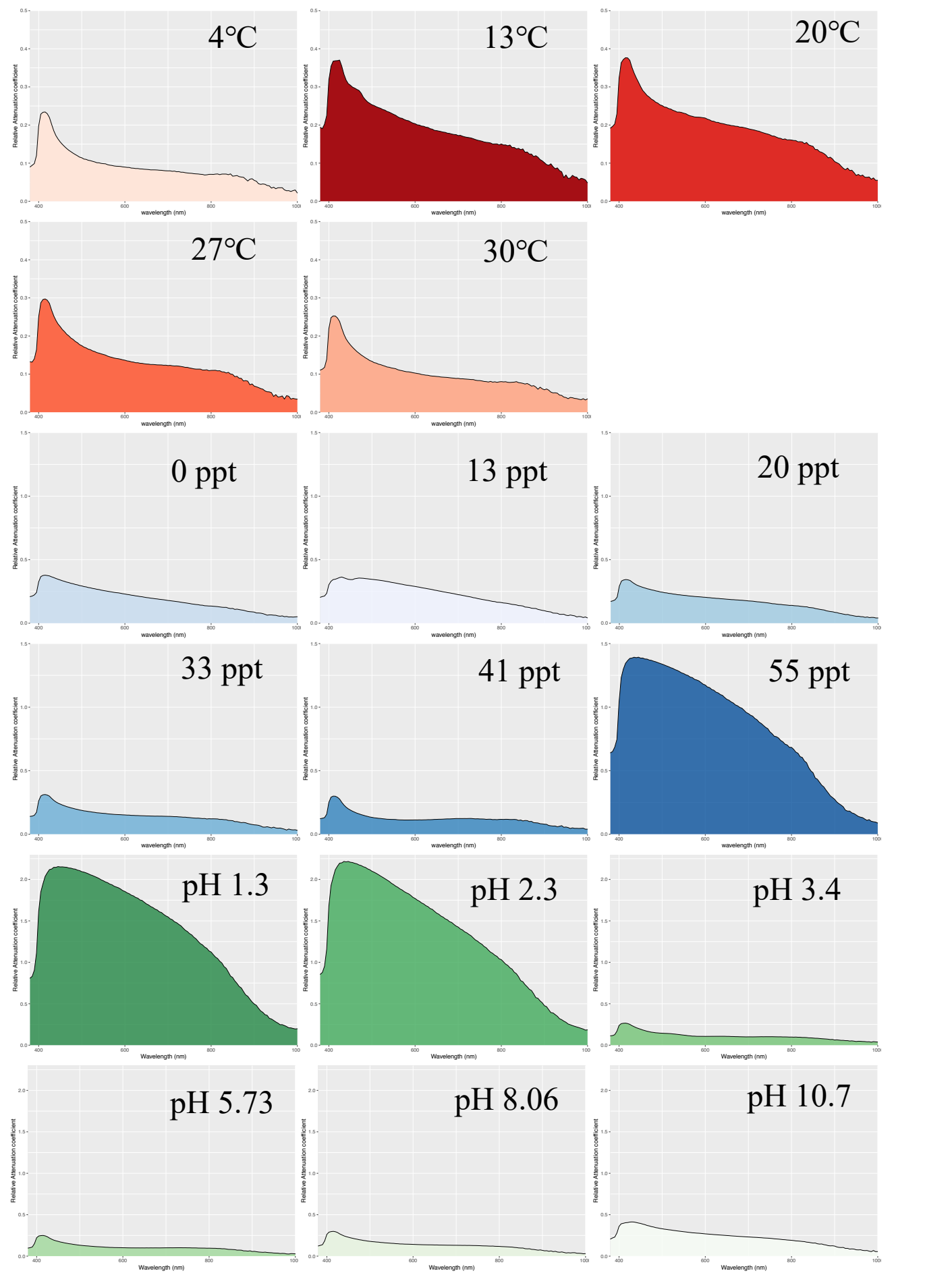

Supplement: S5 Fig — (PDF) [file pone.0292524.s007.pdf]

# S6 Fig.

a

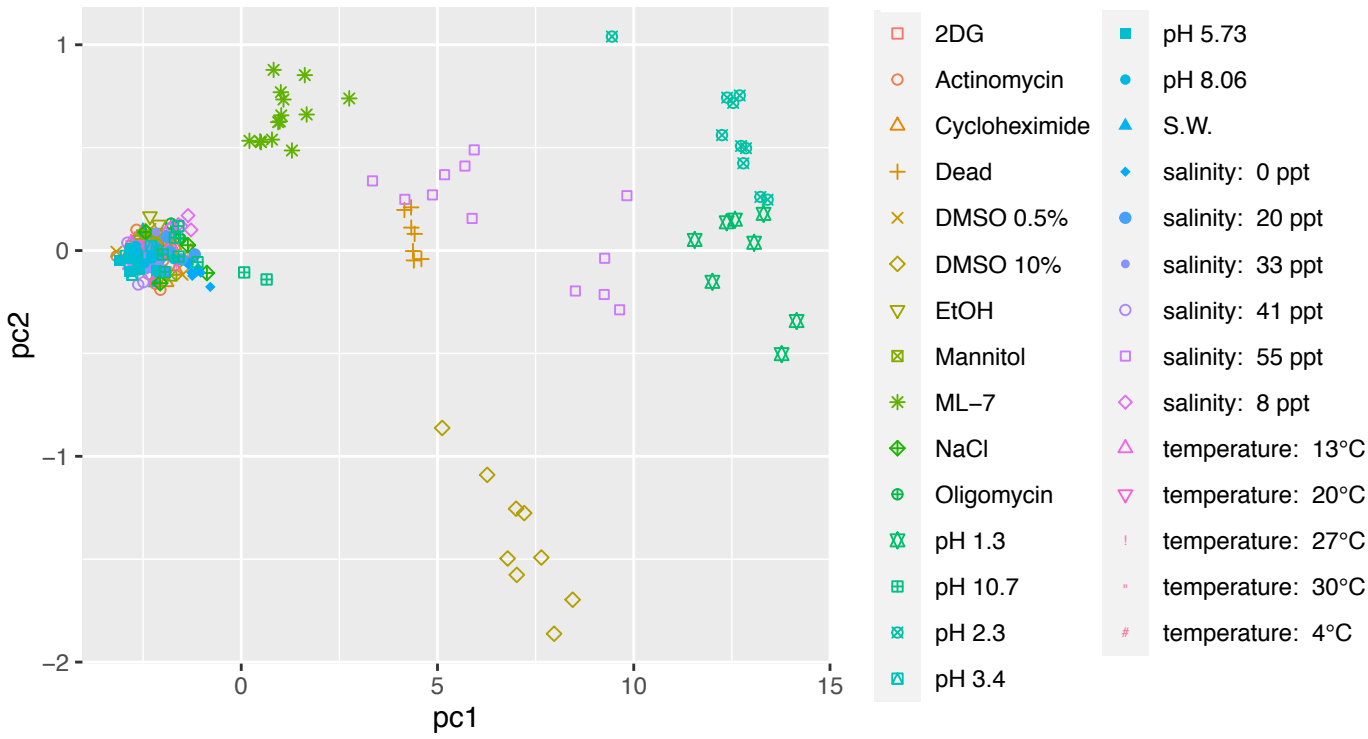

b

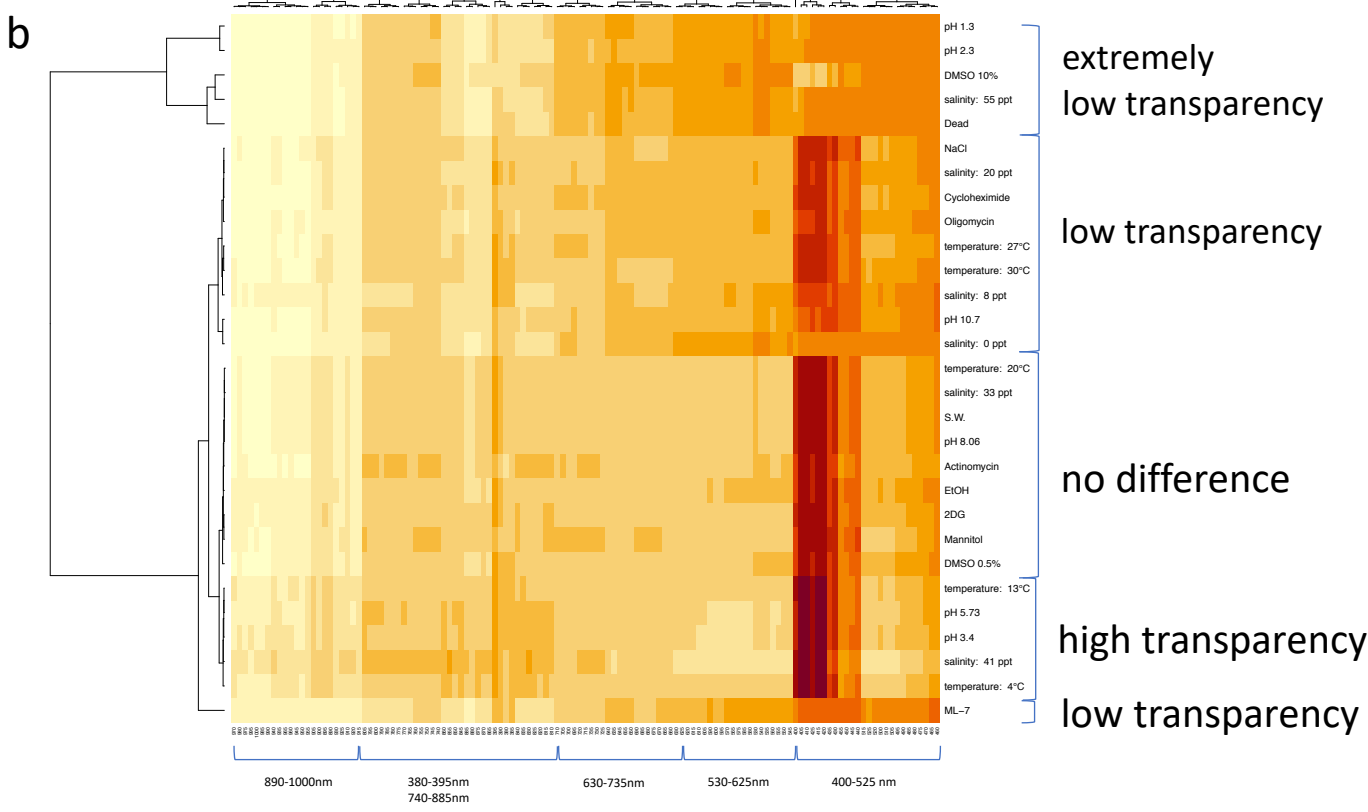

Supplement: S6 Fig — (PDF) [file pone.0292524.s008.pdf]
