# Supplementary material for: Multimodal factor evaluation system for organismal transparency by hyperspectral imaging
Source: PLoS One. 2023 Oct 11;18(10):e0292524. doi: 10.1371/journal.pone.0292524 (PMC10566722; doi:10.1371/journal.pone.0292524)
Supplement: Attachment — Submitted filename: ResponseToReviewers_230823 (1).docx [file pone.0292524.s010.docx]

**Response to editor and reviewers’ comments**

**Our responses are written in red.**

Answer to **Reviewer 1**-

The manuscript 'Multimodal factor evaluation system for organismal transparency by hyperspectral imaging' by Shito et al. describes a method to evaluate the impacts of modulating media conditions and cell physiology on the transparency of the eggs of the ascidian Ascidiella aspersa. The core of the method (hyperspectral transparency measurement) is based on a previous report published in Scientific Reports (Shito et al., 2020) by the same group. Here, they show that various environmental parameters (temperature, salinity, pH and culture media) and pharmacological treatments do modify egg's transparency. Although the biological significance is very preliminary, the proof-of-concept of this approach is achieved.

1. →Thank you very much for your positive comments and many useful suggestions. As you say, biological significance is very preliminary, but we believe this established system could apply to find further insight to organismal transparency. According to your suggestions, we rewrote manuscript and answered all your comments.

Here are minor points I suggest to address in order to improve the quality of the manuscript:

- careful proofreading should be performed since the manuscript is, at times, difficult to read. It seems for example that some words are missing in lines 51 and 76.

2. →Thank you for your sincerely suggestions and checking our English. We corrected mistakes you suggested and rechecked whole manuscript.

- line 83: explain how pH was adjusted.

3. →We added a following sentence “pH levels were adjusted using 2N HCl and 2N NaOH with natural seawater.”.

- the word 'inhibitors' seems inadequate for some of the treatments (mannitol, NaCl,...).

4. →Thank you for your suggestion to our mistake. We change “11 inhibitors” to “11 drugs containing pharmacological inhibitors” overall.

- lines 263-265: the pH of the body fluids or the tunic does not seem relevant regarding the egg's transparency.

5. →We eliminated the sentence “This occurred in spite of the fact that *A. aspersa* has acidic body fluid [20]. Preliminary analysis indicated pH 1.0–2.0 in the tunic. This corresponds to the result of *P. nigra* tunic*,* which is a species in the same family [21].”

- line 284: a potentially interesting outcome of the method and results is that different cellular processes may be involved and that they could impact different regions of the spectrum. The authors could help the reader by highlighting some clear examples.

6. →Thank you for your suggestion. We rewrote the discussion sentence as below:

“Utilizing PCA and clustering analysis in conjunction with hyperspectral imaging, it became evident that diverse cellular processes could underlie the transparency of ascidian eggs. Moreover, these processes might influence distinct segments of spectrums. This phenomenon is exemplified by the spectrum exhibiting a sharp decline at 415 nm due to the myosin light chain kinase inhibitor ML-7, in contrast to the gradual gradient reduction caused by 10% DMSO at the peak of 490 nm (Fig 2a).”

- the authors do not comment on the fact that transparency in normal conditions seems maximal/optimal since none of the conditions robustly increases transparency. Have the authors tried to combine the different conditions that increase transparency (low temperature, high salinity and 2DG)?

7. →Thank you for your comment. Some environmental factors (low temperature, high salinity) show significantly high eggs transparency compared to normal conditions. We are just interested in the maximum/optical condition of ascidian eggs, so we are now exploring it by comparing two species looks have different maximum temperatures. We discussed as below at discussion section. “The species *A. aspersa* inhabits Funka Bay in Japan, characterized by relatively cold waters with temperatures of 4–21°C during the reproductive period [17]. This suggests that a colder environment might be conducive to optimizing egg transparency in this species in Japan. *Phallusia philippinensis* in subtropical seas and typically incubated at 20–25°C [18], displays eggs with remarkably high transparency [14,19]. *A. aspersa* and *P. philippinensis* belong to the same family, Ascidiidae. Investigating the mechanisms that optimize egg transparency in these two closely related species within diverse habitats marked by different temperatures presents an intriguing avenue for further research.”

- the authors take into consideration variation/changes in egg diameter by calculating an 'expansion rate'. However, it is not clear whether this rate is used to adjust the transmittance values that are displayed on the figures. I guess the 'attenuation coefficient' does take this parameter into consideration, but it was overall unclear.

8. → We considered egg diameter in “attenuation coefficient” in Supporting Information. But in the result, we just show transparency in Fig1-3 which are not taking into account for the variation of the egg diameter among experiments. This is because the results obtained from both attenuation factor and transparency do not change in relative transparency order between the compared conditions. We added sentence to clarify it as below “Since bio-transparency does not account for the influence of eggs thickness, we calculated the attenuation coefficient (μ) using the egg thickness (*x)* through the following equations.”

- the measures were made after 21hrs incubation. Could the authors comment on the speed of transparency changes?

9. → Changes in transparency may be related to various factors such as metabolism, gene expression, and structural changes in proteins, and the timescales required for these changes are thought to be different. In this experiment, we needed enough time to consider these together. Therefore, we observed after 21hr to obtain the plateau of the change in the transparency. We also would like to investigate the speed of changes in future, because it may could take further insight into the mechanism of the change of transparency.

Answer to **Reviewer 2**-

The munscript by Shito et al. presented the establishment of the method for evaluation of organsmal tranparency and its utility. The authors demontrated the quantification of the effects of pH, temparatures,salinity, and several inhibitors transparency of ascidian (Ascidiella aspersa) eggs. In particular, they verified that the trasparency mechanisms varied among these environmental or phamacological factors. The methods are appropriate, the results are clear, and the discussion is sufficient. Overall, this manucript is well-written, and the presented method will contribute to advances in investigations of organimal transparency. Consequently, the reviewer makes a few minor comments as below.

10. →Thank you for your sincerely review and positive comments. According to your suggestions, we rewrote manuscript and answered all your comments.

(1) The reviewer wonders whether the ascidian eggs were all vital under experimental conditions. For example, actynomycin D (a transcriptional inhibitor) and cycloheximide (translational inhibitor) frequently causes apoprosis or other cell death. Moreover, such low pH may be harmful to the eggs, while the tunic of the adult ascidians are resistant against it. Otherwise, The reviewer also speculates that the viability of the eggs could be ignored in this research. Please state abot it.

11. →Thank you for your suggestion. The aim of this work is establishing the evaluation system, so we didn’t have additional experiment to investigate the viability of the eggs. We would like to investigate the relationship of viability and organismal transparency by new methods like as measuring the caspase activity. We added sentence as below “The primary focus of this study was to establish an evaluation system; thus, the viability of eggs under different conditions was not within the scope of this research.”

(2) In the Discussion. The authors discuss about carotenoids as pH-sensitive compounds responsible for transparency. The reviewer guesses that Low anf high pH are expected to affect 3-dimentional structures of proteins responsible for transparency (e.g., alpha-crystallin that is a major component of mammalian lens) . Likewise, tmepaerature also affects 3-dimentional structures of proteins, which may also modulate transparency. If possible, please discuss transparency in light of 3-demenotinal structural changes induced by alteration of pH and temperature.

12. →Thank you very much for your useful suggestions. According to your suggestion, we added the text as below “On the other hands, deviations towards low pH levels and raising temperature are likely to impact the three-dimensional structures of proteins accountable for transparency, such as alpha-crystallin—a major component of vertebrate lenses [28]. These variations possibly influence the three-dimensional configuration of some proteins, potentially serving as a modulator of transparency of ascidian eggs.”

(3) Line 30. "suggesting" is better than "which suggested"; Line 240. Insert a space after "ascidian".

13. →Thank you for your correcting. We fixed.
